# Supplementary material for: Analysis of LRRN3, MEF2C, SLC22A, and P2RY12 Gene Expression in the Peripheral Blood of Patients in the Early Stages of Parkinson’s Disease
Source: Biomedicines. 2024 Jun 23;12(7):1391. doi: 10.3390/biomedicines12071391 (PMC11273708; doi:10.3390/biomedicines12071391)
Supplement: Supplementary file 1 [file biomedicines-12-01391-s001.zip › biomedicines-3004159-supplementary.pdf]

biomedicines

MDPI

SUPPLEMENTARY MATERIALS

Supplement Table S1. Characteristics of cohorts of patients with PD and neurological control used in the study.

	Patients with PD		Neurological Control
	Treated	Untreated	
Number of patients	15	13	35
Mean age±SD	62.9±10.5	59.9±11.2	46.1±14.5
Sex ratio	67% females, 33% males	60% females 40% males	67% females 33% males
Other features	Distribution by stages of the Henn-Yahr scale: stage 1-2 - 4 patients, stage 3 - 9 patients, stage 4 - 2 patients.<br><br>Patients with PD who received therapy were treated with different medications with dopamine receptor agonists (pramipexole at a dose of 1.5-3mg/day, L-dopa/carbidopa at a dose of 200–250+25mg/day, or amantadine at a dose of 200-300mg/day) in various combinations	Distribution by stages of the Henn-Yahr scale: stage 1 - 6 patients, stage 2 - 6 patients, stage 1-2 - 1 patient.	The cohort included patients with: Gaucher disease - 5 people, ataxia - 15 people, hepatolenticular degeneration, polyneuropathy - 2 people each, dementia with Lewy bodies, primary progressive aphasia, olivopontocerebellar atrophy, functional movement disorders, impaired glucose tolerance, multiple system atrophy, dermatomyositis, rigid person syndrome, frontotemporal dementia, channelopathy, dystonia - 1 person each. This sample did not include any patients with stroke.

Supplement Table S2. Sequences of gene-specific primers and probes.

Gene	Nucleotide Sequence
SARS1<br>NM_001330669.1 [1]	Probe: 5′-VIC-TCGCCACTCGCTGTCTGCCTTCACCA-BHQ2-3′<br>Forward primer: 5′-CCCAGCCCTCATCCGAGAG-3′<br>Reverse primer: 5′-TGTTCAAGTTGTCTGCCCGAAATC-3′
PSMD6<br>NM_001271779.1	Probe: 5′-VIC-AGGCGGTTTCTCCTGTCCCAGTCTCCTC-BHQ2-3′<br>Forward primer: 5′-AACACAGAAAAGGCCAAAAGCTTAAT-3′<br>Reverse primer: 5′-AATAGCCACACAATAAAGACCCTGAT-3′
LRRN3<br>NM_001099660.2	Probe: 5′-VIC-GCAGGAATAGCAGCATGATGCCAGGG-BHQ2-3′<br>Forward primer: 5′-GGAGAAAGAGAAAGAAGAGGAAGATGT-3′<br>Reverse primer: 5′-GAAGGTGGAGTATGTATAGATGGTCATAG-3′
MEF2C<br>NM_002397.5	Probe: 5′-VIC-TCCACCAGGCAGCAAGAATACGATGC-BHQ2-3′<br>Forward primer: 5′-CCCAATGAATTTAGGAATGAATAACCG-3′<br>Reverse primer: 5′-GTAGCCAATGACTGAGCCGA-3′
SLC22A4<br>NM_003059.3	Probe: 5′-VIC-TCTGACTGTCCTGATTGGAATCCTCACCCT-BHQ2-3′<br>Forward primer: 5′-GAATGCTGCCCTACATCGTCAT-3′<br>Reverse primer: 5′-TTCTTCTGTCTCCATTGAGTCTCTTG-3′
P2RY12<br>NM_022788.5	Probe: 5′-VIC-GACGGCTTGCATTTCTTGTTGGTTACC-BHQ2-3′<br>Forward primer: 5′-GCAATAACTACTACTTACTGGATACATTC-3′<br>Reverse primer: 5′-AAGAGGACCTGGGTGATTTTG-3′

[1] Accession numbers in the NCBI-GenBank database (Release 254.0).

VIC – fluorescent dye, **BHQ2** – fluorescence quencher.

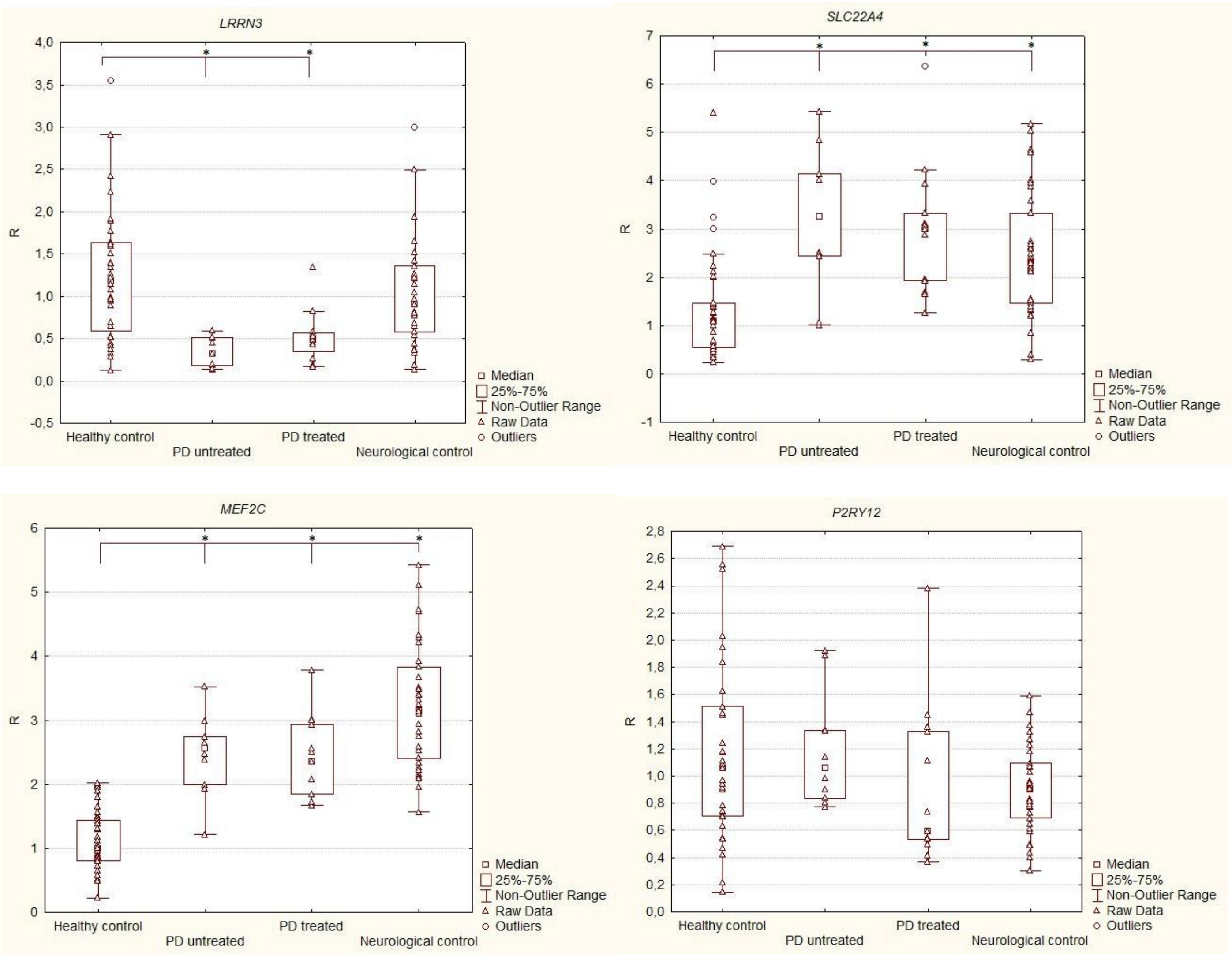

Supplement Figure S1. Results of analysis of changes in the relative levels of mRNA of *LRRN3, MEF2C, SLC22A4,* and *P2RY12* genes in the peripheral blood of patients with early stages of PD. The results (fold change relative to healthy control) are presented as bar plots showing the individual data points. Asterisks show the changes in gene expression significant at $p < 0.05$.
